# Rhabdomyolysis associated with newer-generation anti-seizure medications (ASMs): a real-world retrospective and pharmacovigilance study

**DOI:** 10.3389/fphar.2023.1197470

**Published:** 2023-10-02

**Authors:** Zhenzhen Deng, Shengfeng Wang, Cuifang Wu

**Affiliations:** Department of Pharmacy, The Third Xiangya Hospital, Central South University, Changsha, Hunan, China

**Keywords:** newer-generation anti-seizure medications, rhabdomyolysis, FAERS database, adverse drug reaction, drug safety

## Abstract

**Objective:** Rhabdomyolysis is a potentially fatal adverse reaction mostly triggered by certain medications. Few real-world studies have shown a clear association between newer-generation anti-seizure medications (ASMs) and rhabdomyolysis. We sought to quantify the risk and evaluate the clinical features and management of rhabdomyolysis associated with newer-generation ASMs.

**Methods:** Data were retrieved from the US FDA Adverse Event Reporting System database (FAERS) from 2018 to 2022 on newer-generation ASMs to identify rhabdomyolysis events, and disproportionality analyses were conducted by estimating the reporting odds ratios (RORs) and corresponding 95% confidence intervals (CIs). Furthermore, case reports from 2012 to 31 December 2022 on newer-generation ASMs-induced rhabdomyolysis were retrieved for retrospective analysis.

**Results:** A total of 1,130 rhabdomyolysis reports from the FAERS database were considered. Levetiracetam had the greatest proportion and the highest positive signal values of rhabdomyolysis. The RORs (95% CIs) for newer-generation ASMs were, in descending order, levetiracetam 8.01 (7.26–8.84), lamotrigine 3.78 (3.25–4.40), oxcarbazepine 3.47 (2.53–4.75), pregabalin 2.75 (2.43–3.12), lacosamide 1.85 (1.29–2.65), topiramate 1.64 (1.25–2.15), and gabapentin 1.32 (1.13–1.55). Twenty-six case reports showed evidence of rhabdomyolysis, and levetiracetam (65.4%) was the most frequently reported agent. The median age was 32 years; typical initial symptoms included muscle weakness (34.8%), myalgia (34.8%), backache (17.4%), fatigue (13.0%) and leg pain (8.7%). The median time to onset of rhabdomyolysis was 2 days. All cases had elevated creatine phosphokinase (CPK), and some cases were accompanied by elevated creatinine (57.1%) and myoglobinuria (53.8%). Cessation of ASMs could lead to complete clinical remission. The median time for creatine phosphokinase (CPK) normalization was 8 days.

**Conclusion:** This study identified 7 newer-generation ASMs with significant rhabdomyolysis reporting associations. Prescribers should be more aware of this risk and teach patients to recognize rhabdomyolysis signs/symptoms early.

## Introduction

Rhabdomyolysis is an acute muscle disorder caused by a rapid breakdown of the integrity of skeletal muscle cells, accompanied by the release of electrolytes and muscle cell contents into the blood, causing cellular dysfunction (Cabral et al., 2020). Rhabdomyolysis usually results from traumatic or nontraumatic injury to skeletal muscle and can occur in all age groups and both sexes (Cervellin et al., 2017). The typical signs and symptoms of rhabdomyolysis are called the classic triad: acute or subacute myalgia, muscle weakness mostly in the proximal lower limbs and dark tea-coloured urine (Cervellin et al., 2010). However, more than 50% of patients do not have muscle pain or weakness, and fewer than 10% of patients present with a full triad (Bosch et al., 2009). Clinical manifestations vary from a subclinical elevation of serum creatine phosphokinase (CPK), lactate dehydrogenase (LDH), and aspartate aminotransferase (AST) to severe electrolyte imbalance, cardiac arrhythmia, acute renal failure and disseminated intravascular coagulation (DIC), which depend on the extent of muscle damage (Cabral et al., 2020). A survey indicated that rhabdomyolysis is at least 5 times more likely to be the result of a nontraumatic than traumatic factor (Watson et al., 2014), and drugs are one of the most common causes of rhabdomyolysis in adults. The drugs most often suspected on inducing rhabdomyolysis are statins, fibrates, psychotropic substances, antibiotics and certain herbs (Hohenegger, 2012; Torres et al., 2015). Rhabdomyolysis caused by antiepileptic drugs has become an issue in recent years, although it is still a rare event (Siniscalchi et al., 2021).

Over the past 3 decades, numerous anti-seizure medications (ASMs) with different mechanisms of action have been introduced with the aim of providing better efficacy or safety profiles than the previous drugs, and the older-generation ASMs are increasingly being replaced by the newer-generation ASMs such as levetiracetam, lamotrigine, gabapentin, oxcarbazepine, lacosamide, topiramate, and zonisamide. Research has revealed that the newer generation of ASMs have advantages in terms of drug‒drug interactions, pharmacokinetics and teratogenicity and offer valuable individualized options for the treatment of epilepsy (Chen et al., 2020). Growing evidences from case reports and reviews have suggested that these drugs are associated with rhabdomyolysis (Jiang et al., 2016). However, limited real-world data are available regarding complications related to the newer-generation ASMs-induced rhabdomyolysis, suggesting that this adverse event (AE) may be a neglected risk in therapy with these agents.

In the present study, we searched for signals in the FDA Adverse Event Reporting System (FAERS) database and described the clinical characteristics, management, and prognosis of rhabdomyolysis in patients after newer-generation ASMs therapy in real-life settings, with the goal of raising clinicians’ awareness of this adverse effect and increasing its early diagnosis.

## Materials and methods

### Study design

In this study, a retrospective, disproportionality, pharmacovigilance study was conducted. Data were collected from the publicly released FAERS database on the FDA website from 2018 Quarter 1 (Q1) to 2022 Q3 to assess the risk of rhabdomyolysis from different newer-generation ASMs in a large population. More recent data were chosen because the epidemiology of rhabdomyolysis is changing constantly. Duplicate reports were removed by case number, with only the most recently submitted version included. Reports containing drugs that were administered by oral, intramuscular, subcutaneous, intravenous, and parenteral routes were included, while other routes of administration were excluded.

### Drug exposure and adverse drug reaction definition

The AEs of newer-generation ASMs were encoded by the preferred terms (PTs) in the Medical Dictionary for Regulatory Activities 24.0 (MedDRA). We only used the MedDRA PT “rhabdomyolysis” to identify relevant cases. Other terms, such as “myalgia” and “creatine phosphokinase increased,” were not used because these terms do not guarantee rhabdomyolysis. The newer-generation ASMs were selected from the Drugs@FDA Database and were identified by generic and brand names. Drugs are assigned a role (primary suspect, secondary suspect, concomitant, interacting) by the person reporting the adverse drug reaction (ADR). The newer-generation ASMs which fewer than three rhabdomyolysis ADR reports were excluded from the data analysis.

### Pharmacovigilance study

Disproportionality analysis was employed to detect safety signals by using the reporting odds ratio (ROR). When a target drug is more likely to induce a target AE than all other drugs, it will typically obtain a higher score due to a higher disproportionality. The equations and criteria for the algorithm are shown in Supplementary Table S1. A reporting association was considered to be statistically significant if the lower limit of the 95% CI was >1.0. All data processing and statistical analyses were performed using MySQL 8.0, Navicat Premium 15, Microsoft Excel 2022, and GraphPad Prism 8 (GraphPad Software, CA, United States).

### Descriptive study

A comprehensive search of multiple electronic databases, including PubMed, Embase, Wanfang, China National Knowledge Infrastructure (CNKI) and China Biology Medicine Disc (CBMdisc), from January 2012 to December 2022 regarding newer-generation ASM-induced rhabdomyolysis was conducted, with no language restrictions. The search terms were “levetiracetam,” “lamotrigine,” “pregabalin,” “gabapentin,” “lacosamide,” “topiramate,” “oxcarbazepine,” “perampanel,” “brivaracetam,” “rufinamide,” “rhabdomyolysis,” “myalgia” and “creatine phosphokinase”. We included case reports and excluded preliminary studies, mechanistic studies, animal studies, reviews, duplicate literature and articles with no available full text. Two reviewers searched the literature and collected data independently. Clinical characteristics including age, sex, region of patients, indication, medical history, time to onset, clinical manifestations, laboratory features, treatment and prognosis with newer generation induced rhabdomyolysis were collected. The time to onset of target rhabdomyolysis was defined as from the date of initiation of the antiepileptic drugs to the onset of target rhabdomyolysis data.

## Results

### Newer-generation ASMs-associated rhabdomyolysis in the FAERS database

From January 2018 to September 2022, a total of 261,586 newer-generation ASMs-associated AEs were recorded in the FAERS database, among which 1,142 were for rhabdomyolysis. Levetiracetam was the most reported drug in rhabdomyolysis cases (*n* = 421, 36.9%), followed by pregabalin (*n* = 255, 22.3%), lamotrigine (*n* = 171, 15.0%), gabapentin (*n* = 162, 14.2%), topiramate (*n* = 52, 4.6%), oxcarbazepine (*n* = 39, 3.4%), lacosamide (*n* = 30, 2.6%), perampanel (*n* = 4, 0.4%), zonisamide (*n* = 4, 0.4%), brivaracetam (*n* = 3, 0.3%) and rufinamide (*n* = 1, 0.1%). The reported clinical characteristics of the primary suspected drugs are described in Table 1.

**TABLE 1:** Main characteristics of primary suspect cases of rhabdomyolysis induced by newer-generation ASMs in FAERS.

Variable	N, %
Total number	217
Age	
≤18	23 (10.6)
19–50	117 (53.9)
51–75	30 (13.8)
≥76	6 (2.8)
Unknown	41 (18.9)
Mean age, year (range)	36 (0–90)
Gender	
Female	56 (25.8)
Male	111 (51.2)
Unknown	50 (23.0)
Reporting regions	
Asia	70 (32.3)
Europe	81 (37.3)
North America	58 (26.7)
South America	2 (0.9)
Unknown	6 (2.8)
Reporting year	
2018–2019	83 (38.2)
2019–2020	49 (22.6)
2020–2021	37 (17.1)
2021–2022	48 (22.1)
Newer-generation ASMs	
Levetiracetam	160 (73.7)
Lamotrigine	22 (10.1)
Pregabalin	10 (4.6)
Gabapentin	20 (9.2)
Lacosamide	3 (1.4)
Topiramate	1 (0.5)
Oxcarbazepine	1 (0.5)
Outcome of events	
Death	9 (4.1)
Life-threatening	18 (8.3)
Hospitalization-initial or prolonged	95 (43.8)
Other medical significant condition	92 (43.4)
Unknown	3 (1.4)
Combination of renal injury	
Acute kidney injury	30 (13.8)
Time of onset, day	
Within 1 day	8 (3.7)
1–7	13 (6.0)
7–14	6 (2.8)
>14	5 (2.3)
Unknown	185 (85.3)

ASMs, anti-seizure medications.

### Disproportionality analysis

**FIGURE 1:**
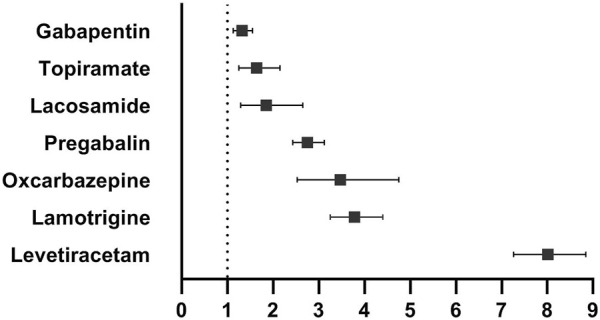
Newer-generation anti-seizure medications (ASMs) and rhabdomyolysis, reporting odds ratios.

Rhabdomyolysis signals for the newer-generation ASMs under the criteria of the ROR are summarized in Table 2. Seven newer-generation ASMs with 1,130 cases involved have been identified as having significant reporting associations with rhabdomyolysis. Levetiracetam had the strongest statistical association with the highest positive signal values of rhabdomyolysis, accounting for 1.14% of all levetiracetam-related reports. Rhabdomyolysis RORs (95% CI) for all newer-generation ASMs were in descending order: levetiracetam 8.01 (7.26–8.84), lamotrigine 3.78 (3.25–4.40), oxcarbazepine 3.47 (2.53–4.75), pregabalin 2.75 (2.43–3.12), lacosamide 1.85 (1.29–2.65), topiramate 1.64 (1.25–2.15), and gabapentin 1.32 (1.13–1.55) (Figure 1).

**TABLE 2:** Counts of rhabdomyolysis with associated ROR for newer-generation ASMs from the FAERS Database.

Drugs	N	ROR (95% two-sided CI)
Levetiracetam	421	8.01 (7.26–8.84)
Lamotrigine	171	3.78 (3.25–4.40)
Oxcarbazepine	39	3.47 (2.53–4.75)
Pregabalin	255	2.75 (2.43–3.12)
Lacosamide	30	1.85 (1.29–2.65)
Topiramate	52	1.64 (1.25–2.15)
Gabapentin	162	1.32 (1.13–1.55)

N, number of adverse events reported; ROR, reporting odds ratio; CI, confidence interval; 95% CI, two-sided for ROR, respectively; ASMs, anti-seizure medications.

### Descriptive analysis

We identified 26 case reports of rhabdomyolysis induced by newer-generation ASMs, with 26 patients included (10 males and 16 females) (Kaufman and Choy, 2012; Gunathilake et al., 2013; Akiyama et al., 2014; Isaacson et al., 2014; Spengler et al., 2014; Incecik et al., 2016; Karaoulanis et al., 2016; Kato et al., 2016; Ramon et al., 2016; Singh et al., 2016; Choi et al., 2017; Coupal et al., 2017; Di Lorenzo and Li, 2017; Kubota et al., 2017; Shahbaz et al., 2017; Matsudaira et al., 2018; Mena-Martin et al., 2018; Rastogi et al., 2018; Rota et al., 2018; Qiu et al., 2019; Thomas et al., 2019; Aslan et al., 2020; Ghosh et al., 2020; Moinuddin, 2020; Alshehabi et al., 2022; Boucher et al., 2022) (Table 3). All the studies were published in English and originated from the following regions: 10 cases from South America, 10 cases from Asia, 5 cases from Europe and 1 case from Oceania. The median age of these patients was 32 years (range 13–75 years), and the majority of patients were between 19 and 50 years old (69.2%), with 3 patients (11.6%) less than 18 years old. Among the newer-generation ASMs, levetiracetam (17 cases, 65.4%) was associated with the highest risk of rhabdomyolysis, followed by gabapentin (4 cases, 15.4%), pregabalin (3 cases, 11.6%), lamotrigine (1 case, 3.8%) and lacosamide (1 case, 3.8%). The concomitant disease was described in 6 cases, including anxiety/depression/schizophrenia (4 cases, 66.7%), hyperlipidemia (4 cases, 66.7%) and hypertension (3 cases, 50.0%). Fourteen patients were taking combinations of medications: other types of ASMs in 6 patients, anti-anxiety/anti-depression drugs in 5 patients, sedatives and hypnotics, cardiovascular drugs and analgesic drugs in 4 patients each. The median time of symptom onset was 2 days (range 1–30 days), with 10 cases (41.7%) occurring within 24 h and 6 cases (25.0%) occurring between 1 and 3 days. The Naranjo scale categorized the probability of newer-generation ASM-induced rhabdomyolysis in the 26 included studies was: 22 cases (84.6%) were classified as probable, 3 cases (11.6%) as possible, and 1 case (3.8%) as doubtful (Table 4).

**TABLE 3:** Newer-generation ASMs related rhabdomyolysis-summary of case reports.

Reference	Region	Drugs	Daily dose (mg)	Age/sex	Concomitant treatment	Initial symptoms	Time of symptom onset	Peak CPK value (U/L)	Peak time (d)	Renal impairment	Urine tested	Other laboratory tests	Therapy	Time to normalization of CPK
Spengler et al. (2014)	United States	levetiracetam	1,000	23/F	Lorazepam	None	1d	1,368	4	Yes	/	/	Drug withdrawal	7d
Akiyama et al. (2014)	Japan	levetiracetam	1,000	29/F	Valproate	Myalgia, backache and weakness	1d	2410	4	/	/	/	Drug withdrawal	28d
Isaacson et al. (2014)	United States	levetiracetam	500	19/M	Oxcarbazepine	None	4d	29,136	8	Yes	Normal	Myoglobin: 228 ng/mL	Drug withdrawal	5d
Incecik et al. (2016)	Turkey	levetiracetam	500	13/F	/	Myalgia	7d	986	7	No	/	Myoglobin: 78 ng/mL	Drug withdrawal	/
Ramon et al. (2016)	France	levetiracetam	1,000	25/M	/	/	3d	15,811	4	/	/	/	Drug withdrawal	3d
Singh et al. (2016)	United States	levetiracetam	1,500	16/M	lorazepam	back pain	1d	15,111	4	Yes	Myoglobinuria	/	Drug withdrawal	9d
Di Lorenzo and Li (2017)	United States	levetiracetam	2,000	27/M	/	None	2d	49,539	5	/	Myoglobinuria	/	Drug withdrawal	38d
Shahbaz et al. (2017)	Pakistan	levetiracetam	1,000	43/M	Topiramate	None	1d	29,750	7	No	/	/	Drug withdrawal	7d
Kubota et al. (2017)	Japan	levetiracetam	500	26/F	Carbamazepine	Weakness	15d	4,396	15	No	/	/	Drug withdrawal	10d
					Phenobarbital									
Rota et al. (2018)	Italy	levetiracetam	1,000	48/F	/	Myalgia	2d	8,199	4	No	/	Myoglobin: 165 ng/mL	Drug withdrawal	6d
Rastogi et al. (2018)	United States	levetiracetam	1,000	42/M	Risperidone	None	1d	30,000	3	Yes	Myoglobinuria	/	Drug withdrawal	5d
Mena-Martin et al. (2018)	Spain	levetiracetam	2,000	28/M	/	None	1d	1,559	7	Yes	/	/	Drug withdrawal	5d
Thomas et al. (2019)	French	levetiracetam	1,000	62/M	/	Myalgia, back pain	2d	19,518	6	No	/	/	Drug withdrawal	8d
Aslan et al. (2020)	Turkey	levetiracetam	1,000	15/M	/	Myalgia, weakness	5d	26,798	2	No	Myoglobinuria	LDH: 501 U/L; Myoglobin: 3,000 ng/mL	Drug withdrawal	5d
Moinuddin. (2020)	United States	levetiracetam	1,000	22/M	/	None	1d	21,936	5	No	Normal	Myoglobin: 594 ng/mL	Drug withdrawal	9d
Alshehabi et al. (2022)	Saudi Arabia	levetiracetam	1,000	36/M	/	Fatigue	730d	921,672	7	Yes	/	/	Hemodialysis	7d
Boucher et al. (2022)	United States	levetiracetam	1,000	35/M	/	None	1d	47,078	5	Yes	Normal	/	Drug withdrawal	/
Chio et al. (2017)	Korea	gabapentin	1,800	32/F	Hydromorphone	Fatigue, weakness	30d	68,680	30	Yes	Myoglobinuria	LDH: 2,200 U/L	Continuous renal replacement	30d
Coupal et al. (2017)	Canada	gabapentin	3,000	31/F	Naproxen	leg pain	21d	44,360	21	No	Normal	LDH: 1,172 U/L	Drug withdrawal	/
					Trazodone									
					Paroxietine	weakness								
					Hydromorphone									
Qiu et al. (2019)	United States	gabapentin	4,200	39/M	Atorvastatin	/	1d	52,800	1	Yes	Normal	Myoglobin: 20,000 ng/mL; LDH: 1,822 U/L	Hemodialysis	21d
Ghosh et al. (2020)	Qatar	gabapentin	1,800	29/M	/	Weakness	1d	747	21	No	/	Myoglobin: 152 ng/mL	Drug withdrawal	14d
Kaufman and Choy. (2012)	United States	pregabalin	300	70/M	Simvastatin	Weakness, leg and back pain	/	14,191	2	Yes	/	/	Drug withdrawal	/
					Oxycodone									
					Amitriptyline									
Gunathilake et al. (2013)	Australia	pregabalin	150	66/F	Atorvastatin	Myalgia and weakness	2d	14,050	5	Yes	Myoglobinuria	/	Drug withdrawal	8d
					Valproic acid									
					Acetaminophen									
					Venlafaxine									
					Oxycodone									
Kato et al. (2016)	Japan	pregabalin	150	75/F	Fenofibrate	Myalgia and weakness	3d	1,250	3	No	Myoglobinuria	/	Drug withdrawal	5d
Karaoulanis et al. (2016)	Greece	lamotrigine	6,000	48/F	Oxcarbazepine	/	/	2,500	5	/	/	/	Drug withdrawal	3d
					Olanzapine									
Matsudaira et al. (2018)	Japan	lacosamide	100	72/M	/	None	15d	1,868	15	/	Normal	LDH: 309 U/L	Drug withdrawal	10d

F, female; M, male; d, day; LDH, lactate dehydrogenase; CPK, creatine phosphokinase; ASMs, anti-seizure medications.

**TABLE 4:** Summary of demographic features of patients with rhabdomyolysis induced by newer-generation ASMs.

Variable	Value (%)
Total	26
Gender	
Female	10 (38.5)
Male	16 (61.5)
Age, years (*n* = 26)	
≤18	3 (11.6)
19–50	18 (69.2)
51–75	5 (19.2)
Median	32 (13–75)
Reporter region (*n* = 26)	
South America	10 (38.5)
Europe	5 (19.2)
Asian	10 (38.5)
Oceania	1 (3.8)
Newer generation ASMs (*n* = 26)	
Pregabalin	3 (11.6)
Gabapentin	4 (15.4)
Lamotrigine	1 (3.8)
Lacosamide	1 (3.8)
levetiracetam	17 (65.4)
Indication (*n* = 24)	
Epilepsy	18 (69.2)
Pain	5 (19.2)
Bipolar disorder	1 (3.8)
Concomitant disease (*n* = 6)	
Anxiety/Depression/Schizophrenia	4 (66.7)
Hyperlipidemia	4 (66.7)
Hypertension	3 (50.0)
Combination medications (*n* = 14)	
Antiepileptic drugs	6 (42.9)
Anti-anxiety/anti-depression drugs	5 (35.7)
Sedatives and hypnotics drugs	4 (28.6)
Cardiovascular drugs	4 (28.6)
Analgetic drugs	4 (28.6)
NASIDs	2 (14.3)
Other suspicious medications (*n* = 4)	
Statins	3 (75.0)
Azithromycin	1 (25.0)
Time of symptom onset, days (*n* = 24)	
≤1	10 (41.7)
1–3	6 (25.0)
3–7	3 (12.5)
>7	5 (20.8)
Median	2 (1–30)
Naranjo Scale (*n* = 26)	
Probable	22 (84.6)
Possible	3 (11.6)
Doubtful	1 (3.8)

ASMs, anti-seizure medications.

Clinical symptoms were described in 23 patients. Muscle weakness (34.8%), myalgia (34.8%), backache (17.4%), fatigue (13.0%) and leg pain (8.7%) were common symptoms in patients with rhabdomyolysis, while 39.1% of the patients had no symptoms. All 26 included patients had elevated creatine phosphokinase (CPK) levels. The median CPK level was 15,461 U/L (range 747–921672 U/L), and the median peak time of CPK was 5 days (range 1–30 days) after administration of the antiepileptic drug. Elevated lactate dehydrogenase (LDH) was reported in 5 patients, with a median value of 1,172 U/L (309–2,200 U/L), and serum myoglobin was elevated in 7 patients with a median value of 228 ng/mL (78–20,000 ng/mL). Twelve patients had renal injury with significantly elevated creatinine out of the 21 patients in whom creatinine was measured (57.1%), while 42.9% had normal creatinine levels. Urine testing was reported in 13 patients, with 9 (69.2%) experiencing myoglobinuria. All 26 included patients stopped the suspected causative drug immediately after developing rhabdomyolysis, 1 of whom underwent continuous renal replacement and 2 had haemodialysis, 88.5% of whom were without further treatment, and all cases achieved resolution of symptoms and complete remission. The time to normalization of CPK was between 3 and 38 days, with 11 patients (50.0%) normalizing within 7 days and 7 patients normalizing between 8 and 14 days. The median time to normalization of CPK was 8 days (range 3–38 days) (Table 5).

**TABLE 5:** Clinical information with rhabdomyolysis induced by newer-generation ASMs.

Variable	Value (%)
Clinical manifestations (*n* = 23)	
None	9 (39.1)
Myalgia	8 (34.8)
Muscle weakness	8 (34.8)
Fatigue	3 (13.0)
Backache	4 (17.4)
Leg pain	2 (8.7)
Peak laboratory values, median	
Peak CPK, U/L (*n* = 26)	15,461 (747–921672)
Peak time of CPK, day (*n* = 26)	5 (1–30)
LDH, U/L (*n* = 5)	1,172 (309–2200)
Myoglobin, ng/mL, U/L (*n* = 7)	228 (78–20000)
ALT, U/L (*n* = 6)	82 (31–220)
AST, U/L (*n* = 7)	157 (38–1,130)
Creatinine, umol/L (*n* = 21)	
Elevated	12 (57.1)
Normal	9 (42.9)
Urine tested (*n* = 13)	
Myoglobinuria	9 (69.2)
Normal	4 (30.8)
Therapy (*n* = 26)	
Drug withdrawal	26 (100)
Continuous renal replacement	1 (3.8)
Hemodialysis	2 (7.7)
Time to normalization of CPK, day (*n* = 22)	
≤7	11 (50.0)
8–14	7 (31.8)
>14	4 (18.2)
Median	8 (3–38)

ALT, alanine aminotransferase; AST, aspartate aminotransferase; CPK, creatine phosphokinase; LDH, lactate dehydrogenase; ASMs, anti-seizure medications.

## Discussion

To the best of our knowledge, this is the largest real-life comparative study reporting rhabdomyolysis after treatment with newer-generation ASMs along with a supportive disproportionality analysis. We performed analysis of the last 57 months of FAERS data and identified 7 newer-generation ASMs that had significant reporting associations with rhabdomyolysis. We found that levetiracetam had the greatest proportion of rhabdomyolysis events, with the highest positive signal values. The other newer-generation ASMs with rhabdomyolysis reports included lamotrigine, oxcarbazepine, pregabalin, lacosamide, topiramate, and gabapentin. In the descriptive analysis, levetiracetam was the most frequently reported potential agent for rhabdomyolysis as well, accounting for 65.4% of all cases included. Currently, only pregabalin lists rhabdomyolysis as a possible adverse reaction in its package insert. Our findings are clinically relevant because they will be helpful to improve clinicians’ awareness, increase early diagnosis and guide clinical treatment of newer-generation ASM–induced rhabdomyolysis. In addition, we provide separate analyses of initial symptom onset, laboratory features, treatment, outcomes, and prognosis, which had not been done in previous studies. By collecting medical records from published case reports involving newer-generation ASM-associated rhabdomyolysis, we found that muscle weakness, myalgia, backache, fatigue and leg pain were the most common symptoms, which were often accompanied by elevated CPK, LDH, creatinine, and serum myoglobin. Therefore, it is recommended that patients with muscle weakness, myalgia, or pain be alerted to the possibility of rhabdomyolysis and undergo regular laboratory tests during newer-generation ASM treatment.

We observed that newer-generation ASMs can cause rhabdomyolysis early in treatment, with a median onset time of 2 days (range 1–30 days), and approximately 66.7% of the included cases occurred within 3 days, indicating that suspicious symptoms and laboratory indicators associated with rhabdomyolysis should be monitored early in treatment in particular. The timing of the onset of rhabdomyolysis varied depending on the type of medication involved. A previous post-marketing analysis from the FAERS data raised a safety signal detecting 48 reports of LEV-induced rhabdomyolysis from 2004 to 2015, which had a median time to onset of a few days (Carnovale et al., 2017). This interval is comparable to that of rhabdomyolysis induced by other drugs, such as antibiotics, particularly quinolone (Bouchard et al., 2019), but shorter than in paradoxical cases reported with statins (Vinci et al., 2021), with which it occurs within a few weeks to months. There was one report of a 36-year-old male who took levetiracetam for 2 years before rhabdomyolysis onset, whom we did not include in the calculation of the median onset time to avoid negative skewing of the results (Alshehabi et al., 2022). It should be noted that the intake of high doses of ASMs can be the cause of myotoxicity. We observed that three of the four patients on gabapentin therapy developed rhabdomyolysis immediately after the dose increased, and 2 patients exceeded the maximum dosage recommended by the package insert. Considering the limited number of cases here, the relationship between the occurrence of rhabdomyolysis and the daily dose of newer-generation ASMs needs to be further confirmed by prospective studies. In addition, we observed that 4 patients took combination therapies that may cause rhabdomyolysis, including statins (3 cases) and azithromycin (1 case) (Kato et al., 2016). Rhabdomyolysis is known to be a common adverse effect of statins. Of the three patients who took statins as concomitant therapy, two patients took statins for a longer period (Gunathilake et al., 2013; Qiu et al., 2019), and one patient developed myalgia symptoms after 4 days of statin and increased dose of gabapentin for 1 day. In this case, both statins and gabapentin may have been responsible for his rhabdomyolysis (Kaufman and Choy, 2012).

It remains difficult to demonstrate whether certain ASMs are triggering factors or happen to be present in rhabdomyolysis cases. In some cases of rhabdomyolysis, drug interactions may have played a potential role due to either altered bioavailability and/or decreased clearance (Duncan and Howden, 2017). Proton pump inhibitors (PPIs) can inhibit the cytochrome P450 enzyme system and increase the bioavailability of statins to increase the risk of statin-related rhabdomyolysis (Clark and Strandell, 2006). However, *in vitro* studies of pregabalin, gabapentin, and levetiracetam have shown that they do not inhibit drug metabolism and have low plasma protein binding rates, meaning they rarely interact with other drugs. Lamotrigine also showed no evident hepatic enzyme induction and had no correlation with drugs metabolized by cytochrome P450 enzymes. Therefore, we tend to believe that the newer-generation ASMs themselves act as triggering factors for rhabdomyolysis, after ruling out possible drug interactions. It is worth noting that genetic polymorphisms relevant to pharmacokinetics (e.g., in drug receptors, transporters and metabolizing enzymes) and pharmacodynamics predispose patients to myopathy. It has been validated that polymorphisms in the SLCO1B1 gene, which encodes the protein responsible for hepatic uptake of statins, and the COQ2 gene, which encodes an enzyme involved in the synthesis of coenzyme Q10, are strongly associated with statin-induced myopathy (Needham and Mastaglia, 2014). Recently, new ASMs that have been investigated in populations across the world and found to be significantly affected by metabolic enzymes and their genetic polymorphisms include lamotrigine, oxcarbazepine and levetiracetam (Zhao and Meng, 2022). Unfortunately, there is no clear evidence for the relationship between genetic polymorphisms affecting new AEDs and rhabdomyolysis. In the future, investigating genetic factors predisposing to AED-induced rhabdomyolysis might act as the first step towards pharmacogenomic screening to identify at-risk individuals.

The mechanism by which certain ASMs can cause rhabdomyolysis have not been illustrated. In recent years, some studies have used machine learning methods to establish quantitative structure-activity relationship models. Based on the chemical structure information of drugs, the model can well predict drug-induced rhabdomyolysis and its severity, which suggests that the drugs causing rhabdomyolysis might have specific chemical structure (Cui et al., 2019; Zho et al., 2021). It provides a reasonable explanation for different types of newer-generation ASM-associated rhabdomyolysis. The exploration of molecular mechanisms targeting these specific chemical structures will help us to accurately discover the pathogenesis of their muscular toxicity. As for the molecular mechanism, LEV was reported to bind specifically with the SV2A protein, which is a membrane protein specifically expressed in synaptic vesicles and causes neuronal inhibition (Tokudome et al., 2018). However, SV2A was shown to be selectively localized in motor nerve terminals on slow (type I and small type IIA) muscle fibres in mice as well. The receptor present in the muscle fibres may suggest a mechanism for LEV-induced rhabdomyolysis (Chakkalakal et al., 2010). In addition, some research suggests that ferroptosis is a key causative factor in a variety of skeletal muscle diseases, including sarcopenia, rhabdomyolysis, rhabdomyosarcoma, and exhaustive exercise-induced fatigue (Wang et al., 2022). Discovered that ferroptosis serves as a mechanism in statin-induced myopathy. Among four candidates investigated, including atorvastatin, lovastatin, rosuvastatin, and pravastatin, only atorvastatin inhibits human cardiomyocyte (HCM) and murine skeletal muscle cell (C2C12) viability in a dose-dependent manner and leads to ferroptosis in HCM and C2C12 cells (Zhang et al., 2022). As a promising treatment strategy, ferroptosis can be explored for newer-generation ASM-associated rhabdomyolysis in future research.

Optimal management of newer-generation ASM-associated rhabdomyolysis depends on the recognition and elimination of the underlying cause when identifiable. Treatment consists of discontinuing the offending agent and aggressive fluid resuscitation with isotonic saline, which aims to maximize renal perfusion and thus limit intratubular cast formation. We observed that acute kidney injury occurred in 57.1% of rhabdomyolysis patients, who had a median creatinine level of 2.2 mg/dL (range 0.7–13.4 mg/dL), and the patient who had the highest peak CPK developed the most severe AKI, with a peak creatinine level of 1,185 μmol/L (Alshehabi et al., 2022). Research has shown that the mortality rate is 42% in those who develop acute renal failure (Russell, 2005), that any delay in fluid resuscitation increases the risk of acute kidney injury (AKI), and that forced diuresis within 6 h of presentation may reduce the risk of AKI (Zager, 1996; Hebert et al., 2023). Therefore, aggressive intravenous fluid resuscitation is necessary to reduce the damaging effects or prevent a fatal outcome regardless of the presence of AKI. Generally, the prognosis mainly depends on the complications presented and their underlying causes. In our descriptive analysis, the rhabdomyolysis related to newer-generation ASMs was reversible, and the prognosis was good, all patients achieving complete clinical recovery, even those with severe AKI.

## Conclusion

Our retrospective analysis identified 7 newer-generation ASMs with significant reporting associations with rhabdomyolysis: levetiracetam, lamotrigine, oxcarbazepine, pregabalin, lacosamide, topiramate and gabapentin. Physicians and clinical pharmacists should pay close attention to possible clinical symptoms such as muscle weakness, myalgia, backache and fatigue as well as rhabdomyolysis markers like elevated CPK, LDH, creatinine and serum myoglobin during newer-generation ASMs treatment to identify early warning signs and prevent any severe complications. Early evaluation and withdrawal of the offending agent immediately improved the clinical symptoms. Aggressive intravenous fluid resuscitation is necessary to reduce the damaging effects whether or not AKI had occurred. Further prospective studies evaluating risk factors for rhabdomyolysis and concomitant therapies of patients treated with newer-generation ASMs are needed to confirm our findings.

## Data Availability

The original contributions presented in the study are included in the article/Supplementary Material, further inquiries can be directed to the corresponding author.

## References

[B1] Akiyama H. Haga Y. Sasaki N. Yanagisawa T. Hasegawa Y. (2014). A case of rhabdomyolysis in which levetiracetam was suspected as the cause. Epilepsy Behav. Case Rep. 2, 152–155. 10.1016/j.ebcr.2014.08.001 25667895 PMC4308062

[B2] Alshehabi K. M. Askandarani S. Alkhalifah Z. A. (2022). Suspected levetiracetam-induced acute rhabdomyolysis in a patient with retinoblastoma: A case report and literature review. Cureus 14, e25183. 10.7759/cureus.25183 35747052 PMC9209403

[B3] Aslan N. Yildizdas D. Huseyinli B. Horoz O. O. Mert G. G. Ekinci F. (2020). Levetiracetam treatment-associated acute rhabdomyolysis in an adolescent. J. Pediatr. Intensive Care 9, 139–140. 10.1055/s-0039-1700951 32351770 PMC7186017

[B4] Bosch X. Poch E. Grau J. M. (2009). Rhabdomyolysis and acute kidney injury. N. Engl. J. Med. 361, 62–72. 10.1056/NEJMra0801327 19571284

[B5] Bouchard J. De La Pena N. Oleksiuk L. M. (2019). Levofloxacin-induced rhabdomyolysis in a patient on concurrent atorvastatin: Case report and literature review. J. Clin. Pharm. Ther. 44, 966–969. 10.1111/jcpt.13010 31407828

[B6] Boucher K. S. Dedhia N. Bommisetty D. (2022). Levetiracetam-induced rhabdomyolysis following medication Re-initiation. Cureus 14, e30042. 10.7759/cureus.30042 36381889 PMC9637409

[B7] Cabral B. Edding S. N. Portocarrero J. P. Lerma E. V. (2020). Rhabdomyolysis. Dis. Mon. 66, 101015. 10.1016/j.disamonth.2020.101015 32532456

[B8] Carnovale C. Gentili M. Antoniazzi S. Clementi E. Radice S. (2017). Levetiracetam-induced rhabdomyolysis: Analysis of reports from the food and drug administration's adverse event reporting system database. Muscle Nerve 56, E176-E178–8. 10.1002/mus.25972 28888059

[B9] Cervellin G. Comelli I. Benatti M. Sanchis-Gomar F. Bassi A. Lippi G. (2017). Non-traumatic rhabdomyolysis: Background, laboratory features, and acute clinical management. Clin. Biochem. 50, 656–662. 10.1016/j.clinbiochem.2017.02.016 28235546

[B10] Cervellin G. Comelli I. Lippi G. (2010). Rhabdomyolysis: historical background, clinical, diagnostic and therapeutic features. Clin. Chem. Lab. Med. 48, 749–756. 10.1515/CCLM.2010.151 20298139

[B11] Chakkalakal J. V. Nishimune H. Ruas J. L. Spiegelman B. M. Sanes J. R. (2010). Retrograde influence of muscle fibers on their innervation revealed by a novel marker for slow motoneurons. DEVELOPMENT 137, 3489–3499. 10.1242/dev.053348 20843861 PMC2947760

[B12] Chen Z. Brodie M. J. Kwan P. (2020). What has been the impact of new drug treatments on epilepsy? Curr. Opin. Neurol. 33, 185–190. 10.1097/WCO.0000000000000803 32049739

[B13] Choi M. S. Jeon H. Kim H. S. Jang B. H. Lee Y. H. Park H. S. (2017). A case of gabapentin-induced rhabdomyolysis requiring renal replacement therapy. Hemodial. Int. 21, E4-E8–E8. 10.1111/hdi.12458 27389284

[B14] Clark D. W. Strandell J. (2006). Myopathy including polymyositis: a likely class adverse effect of proton pump inhibitors? Eur. J. Clin. Pharmacol. 62, 473–479. 10.1007/s00228-006-0131-1 16758264

[B15] Coupal T. M. Chang D. R. Pennycooke K. Ouellette H. A. Munk P. L. (2017). Radiologic findings in gabapentin-induced myositis. J. Radiol. Case Rep. 11, 30–37. 10.3941/jrcr.v11i4.3092 28567183 PMC5439453

[B16] Cui X. Liu J. Zhang J. Wu Q. Li X. (2019). *In silico* prediction of drug-induced rhabdomyolysis with machine-learning models and structural alerts. J. Appl. Toxicol. 39, 1224–1232. 10.1002/jat.3808 31006880

[B17] Di Lorenzo R. Li Y. (2017). Rhabdomyolysis associated with levetiracetam administration. Muscle Nerve 56, E1-E2–E2. 10.1002/mus.25548 28039868

[B18] Duncan S. J. Howden C. W. (2017). Proton pump inhibitors and risk of rhabdomyolysis. Drug Saf. 40, 61–64. 10.1007/s40264-016-0473-2 27838824

[B19] Ghosh S. Villan S. Al Y. W. (2020). Gabapentin-induced myositis in a patient with spinal cord injury - a case report. Qatar Med. J. 2020, 30. 10.5339/qmj.2020.30 33282714 PMC7684549

[B20] Gunathilake R. Boyce L. E. Knight A. T. (2013). Pregabalin-associated rhabdomyolysis. Med. J. Aust. 199, 624–625. 10.5694/mja13.10769 24182230

[B21] Hebert J. F. Burfeind K. G. Malinoski D. Hutchens M. P. (2023). Molecular mechanisms of rhabdomyolysis-induced kidney injury: From bench to bedside. Kidney Int. Rep. 8, 17–29. 10.1016/j.ekir.2022.09.026 36644345 PMC9831947

[B22] Hohenegger M. (2012). Drug induced rhabdomyolysis. Curr. Opin. Pharmacol. 12, 335–339. 10.1016/j.coph.2012.04.002 22560920 PMC3387368

[B23] Incecik F. Herguner O. M. Besen S. Altunbasak S. (2016). Acute rhabdomyolysis associated with levetiracetam therapy in a child. Acta Neurol. Belg 116, 369–370. 10.1007/s13760-015-0542-9 26399431

[B24] Isaacson J. E. Choe D. J. Doherty M. J. (2014). Creatine phosphokinase elevation exacerbated by levetiracetam therapy. Epilepsy Behav. Case Rep. 2, 189–191. 10.1016/j.ebcr.2014.09.008 25667904 PMC4308055

[B25] Jiang W. Wang X. Zhou S. (2016). Rhabdomyolysis induced by antiepileptic drugs: characteristics, treatment and prognosis. EXPERT Opin. DRUG Saf. 15, 357–365. 10.1517/14740338.2016.1139572 26750987

[B26] Karaoulanis S. E. Syngelakis M. Fokas K. (2016). Rhabdomyolysis after lamotrigine overdose: a case report and review of the literature. Ann. Gen. Psychiatry 15, 6. 10.1186/s12991-016-0093-3 26913053 PMC4765213

[B27] Kato K. Iwasaki Y. Onodera K. Higuchi M. Kato Y. (2016). Pregabalin- and azithromycin-induced rhabdomyolysis with purpura: An unrecognized interaction: a case report. Int. J. Surg. Case Rep. 26, 221–223. 10.1016/j.ijscr.2016.07.007 27521491 PMC4983139

[B28] Kaufman M. B. Choy M. (2012). Pregabalin and simvastatin: first report of a case of rhabdomyolysis. P T 37, 579–595. 23115467 PMC3474439

[B29] Kubota K. Yamamoto T. Kawamoto M. (2017). Levetiracetam-induced rhabdomyolysis: A case report and literature review. Neurol. Asia 22 (3), 275–278.

[B30] Matsudaira T. Terada T. Araki Y. Ikeda H. Obi T. Inoue Y. (2018). HyperCKemia associated with lacosamide therapy in an elderly patient with focal onset epilepsy. Seizure 63, 14–16. 10.1016/j.seizure.2018.10.005 30391661

[B31] Mena-Martin F. Gutierrez-Garcia A. Martin-Escudero J. Fernandez-Arconada O. (2018). Acute kidney injury and creatine kinase elevation after beginning treatment with levetiracetam. Eur. Neurol. Rev. 13, 113. 10.17925/enr.2018.13.2.113

[B32] Moinuddin I. A. (2020). Suspected levetiracetam-induced rhabdomyolysis: A case report and literature review. Am. J. Case Rep. 21, e926064. 10.12659/AJCR.926064 33112844 PMC7603803

[B33] Needham M. Mastaglia F. L. (2014). Statin myotoxicity: a review of genetic susceptibility factors. Neuromuscul. Disord. 24, 4–15. 10.1016/j.nmd.2013.09.011 24176465

[B34] Qiu X. Tackett E. Khitan Z. (2019). A case of gabapentin overdose induced rhabdomyolysis requiring renal replacement therapy. Clin. Case Rep. 7, 1596–1599. 10.1002/ccr3.2302 31428399 PMC6692987

[B35] Ramon M. Tourteau E. Lemaire N. Gautier S. Bene J. (2016). HyperCKemia induced by levetiracetam. PRESSE Med. 45, 943–944. 10.1016/j.lpm.2016.05.014 27374267

[B36] Rastogi V. Singh D. Kaur B. Arora P. Gadikota J. P. (2018). Rhabdomyolysis: A rare adverse effect of levetiracetam. Cureus 10, e2705. 10.7759/cureus.2705 30062079 PMC6063379

[B37] Rota E. Arena L. Celli L. Testa L. Morelli N. (2018). Levetiracetam-induced rhabdomyolysis: the first Italian case. Neurol. Sci. 39, 1629–1630. 10.1007/s10072-018-3421-3 29696399

[B38] Russell T. A. (2005). Acute renal failure related to rhabdomyolysis: pathophysiology, diagnosis, and collaborative management. Nephrol. Nurs. J. 32 (409-17), 409–417. 16180782

[B39] Shahbaz N. Younus S. M. Khan S. A. Ain Q. Khan M. A. Memon M. H. (2017). Levetiracetam induced increase in creatine phosphokinase levels. J. Coll. Physicians Surg. Pak 27, S63-S64. 28302251

[B40] Singh R. Patel D. R. Pejka S. (2016). Rhabdomyolysis in a hospitalized 16-year-old boy: A rarely reported underlying cause. Case Rep. Pediatr. 2016, 7873813. 10.1155/2016/7873813 27895953 PMC5118508

[B41] Siniscalchi A. Mintzer S. De Sarro G. Gallelli L. (2021). Myotoxicity induced by antiepileptic drugs: Could be a rare but serious adverse event? Psychopharmacol. Bull. 51, 105–116. 34887602 10.64719/pb.4421 PMC8601760

[B42] Spengler D. C. Montouris G. D. Hohler A. D. (2014). Levetiracetam as a possible contributor to acute kidney injury. Clin. Ther. 36, 1303–1306. 10.1016/j.clinthera.2014.06.002 24986483

[B43] Thomas L. Mirza M. Shaikh N. A. Ahmed N. (2019). Rhabdomyolysis: a rare adverse effect of levetiracetam. BMJ Case Rep. 12, e230851. 10.1136/bcr-2019-230851 PMC6720964 31451475

[B44] Tokudome K. Shimizu S. Serikawa T. Ohno Y. (2018). Function of synaptic vesicle protein 2A (SV2A) as a novel therapeutic target for epilepsy. Nihon Yakurigaku Zasshi 152, 275–280. 10.1254/fpj.152.275 30531097

[B45] Torres P. A. Helmstetter J. A. Kaye A. M. Kaye A. D. (2015). Rhabdomyolysis: pathogenesis, diagnosis, and treatment. Ochsner J. 15, 58–69. 25829882 PMC4365849

[B46] Vinci P. Panizon E. Tosoni L. M. Cerrato C. Pellicori F. Mearelli F. (2021). Statin-associated myopathy: Emphasis on mechanisms and targeted therapy. Int. J. Mol. Sci. 22, 11687. 10.3390/ijms222111687 34769118 PMC8583847

[B47] Wang Y. Zhang Z. Jiao W. Wang X. Zhao Y. (2022). Ferroptosis and its role in skeletal muscle diseases. Front. Mol. Biosci. 9, 1051866. 10.3389/fmolb.2022.1051866 36406272 PMC9669482

[B48] Watson J. D. Gifford S. M. Clouse W. D. (2014). Biochemical markers of acute limb ischemia, rhabdomyolysis, and impact on limb salvage. Semin. Vasc. Surg. 27, 176–181. 10.1053/j.semvascsurg.2015.01.007 26073828

[B49] Zager R. A. (1996). Rhabdomyolysis and myohemoglobinuric acute renal failure. KIDNEY Int. 49, 314–326. 10.1038/ki.1996.48 8821813

[B50] Zhang Q. Qu H. Chen Y. Luo X. Chen C. Xiao B. (2022). Atorvastatin induces mitochondria-dependent ferroptosis via the modulation of nrf2-xCT/GPx4 Axis. Front. Cell Dev. Biol. 10, 806081. 10.3389/fcell.2022.806081 35309902 PMC8927716

[B51] Zhao W. Meng H. (2022). Effects of genetic polymorphism of drug-metabolizing enzymes on the plasma concentrations of antiepileptic drugs in Chinese population. BIOENGINEERED 13, 7709–7745. 10.1080/21655979.2022.2036916 35290166 PMC9278974

[B52] Zhou Y. Li S. Zhao Y. Guo M. Liu Y. Li M. (2021). Quantitative structure-activity relationship (QSAR) model for the severity prediction of drug-induced rhabdomyolysis by using random forest. Chem. Res. Toxicol. 34, 514–521. 10.1021/acs.chemrestox.0c00347 33393765

