# Supplementary material for: Rhabdomyolysis associated with newer-generation anti-seizure medications (ASMs): a real-world retrospective and pharmacovigilance study
Source: Front Pharmacol. 2023 Oct 2;14:1197470. doi: 10.3389/fphar.2023.1197470 (PMC10577175; doi:10.3389/fphar.2023.1197470)
Supplement: Supplementary file 1 [file Table1.docx]

**Supplementary Table S1**. The calculation method and formulas of the reporting odds ratio (ROR).

|  | ADEs of interests | All other ADEs of interests |
| --- | --- | --- |
| Drugs of interests | a | b |
| All other drugs of interests | c | d |

ADE: adverse drug events.

Calculation formulas for ROR:

1. ROR= (a*d)/(b*c)
2. 95%CI=e^ln(ROR)±1.96(1/a+1/b+1/c+1/d)^0.5^
